# Sequence-specific interactions of Rep proteins with ssDNA in the AT-rich region of the plasmid replication origin

**DOI:** 10.1093/nar/gku453

**Published:** 2014-05-16

**Authors:** Katarzyna Wegrzyn, Maria Eugenia Fuentes-Perez, Katarzyna Bury, Magdalena Rajewska, Fernando Moreno-Herrero, Igor Konieczny

**Affiliations:** 1 Department of Molecular and Cellular Biology, Intercollegiate Faculty of Biotechnology, University of Gdansk, 24 Kladki, 80-822 Gdansk, Poland; 2 Department of Macromolecular Structures, Centro Nacional de Biotecnologia, CSIC, Darwin 3, 28049 Cantoblanco, Madrid, Spain

## Abstract

The DNA unwinding element (DUE) is a sequence rich in adenine and thymine residues present within the origin region of both prokaryotic and eukaryotic replicons. Recently, it has been shown that this is the site where bacterial DnaA proteins, the chromosomal replication initiators, form a specific nucleoprotein filament. DnaA proteins contain a DNA binding domain (DBD) and belong to the family of origin binding proteins (OBPs). To date there has been no data on whether OBPs structurally different from DnaA can form nucleoprotein complexes within the DUE. In this work we demonstrate that plasmid Rep proteins, composed of two Winged Helix domains, distinct from the DBD, specifically bind to one of the strands of ssDNA within the DUE. We observed nucleoprotein complexes formed by these Rep proteins, involving both dsDNA containing the Rep-binding sites (iterons) and the strand-specific ssDNA of the DUE. Formation of these complexes required the presence of all repeated sequence elements located within the DUE. Any changes in these repeated sequences resulted in the disturbance in Rep-ssDNA DUE complex formation and the lack of origin replication activity *in vivo* or *in vitro*.

## INTRODUCTION

**Figure 1.**
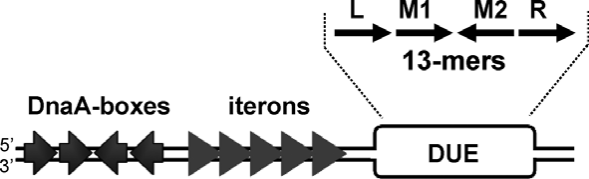
Organization of the RK2 origin region. The scheme presents the RK2 plasmid origin region (*oriV*), which contains DnaA protein binding sites (DnaA-boxes), plasmid replication initiator binding sites (iterons) and an AT-rich region with DUE. Within the DUE, four 13-mer repeats can be identified (marked as black arrows). DnaA-box sequences are depicted as gray arrows; iterons as gray triangles. The origin region is not drawn to scale.

The origins of all replicons contain a region whose sequence is rich in adenine and thymine residues (AT-rich). This site, also named DUE (DNA unwinding element), is the place where the initial destabilization (opening) of the double-stranded DNA (dsDNA) occurs and the replication complex is assembled (1). The opening of the duplex in the DUE creates single-stranded DNA (ssDNA), which is critical for replication initiation. The specificity of the sequence within the DUE element has been analyzed both in prokaryotic and eukaryotic replicons (2–5). In the *Escherichia coli* replication origin (*oriC*), the AT-rich region contains three repeats of 13-nucleotides with the consensus sequence GATCTnTTnnTTT, named left, middle and right (L, M, R), based on their position within the origin (6). The presence of all three repeats is required for the activity of the origin (1). Similar repeats were also identified in plasmid origins. In the replication origin of the broad-host-range RK2 plasmid (*oriV*) there are four 13-mers, L, M1, M2 and R, located in the DUE (Figure 1). Their sequence (consensus TAAACnTTnTTTT) and specific position are crucial for origin activity and affect origin opening and helicase loading (7–9). In the origin of the F plasmid, *oriS*, four repeated sequences within the AT-rich region have been identified (10). They are shorter (8-mers) (consensus TTTTTA^G^/_T_A) in comparison to the 13-mers of *oriC* and *oriV* (11). Repeated motifs of different sequence, length and spacing were also found in the AT-rich region of other plasmids’ origins (e.g. *ori*γ of R6K plasmid (12), *oriR* of R1 (10), *ori* of bacteriophage λ (13) and many others (11)).

The AT-rich region is bound by a number of proteins, either as ssDNA or dsDNA. Some proteins modify the origin's architecture by binding to dsDNA (e.g. IHF (14–16), Fis (17)); others bind to ssDNA and play crucial roles during DNA replication (e.g. DnaB (18–20), DnaG (21,22), PolIII holoenzyme (23)) or by binding to dsDNA in the regulation of this process (e.g. SeqA (24,25), IciA (10,26,27), ArcA (28), HobH (29)). Nevertheless, the main role of this region is to provide a structural scaffold for the assembly of the replication complex. The formation of the ssDNA scaffold for replication proteins is mediated by origin binding proteins (OBPs), which recognize and bind specific sequences within the replicon's origin, close to the DUE element (30). Recent results have shown that the bacterial OBP, DnaA protein, apart from binding specific dsDNA sequences in the origin (the DnaA-boxes), also binds ssDNA DUE (31–34). This sequence-specific interaction with the ssDNA is essential for the opening of the origin and replication activity. Crystallographic data revealed that the DnaA protein forms a helical structure on the ssDNA region close to the nearest DnaA-box (32). For the chromosomally encoded bacterial OBPs the interaction with dsDNA is mediated by a DNA binding domain (DBD) containing helix-turn-helix motif (35) (the exact amino acid sequence of the domain in *E. coli* DnaA protein was determined by mutagenesis (36) and crystallography (37)), which, to date, has not been identified in other, non-chromosomal, replication initiators. It was proposed that during dsDNA binding by the DnaA protein the DBD domain extends away from the body of the protein and exposes its helix-turn-helix motif (31). In contrast, the interaction of chromosomally encoded bacterial OBPs with ssDNA is mediated by a central AAA+ domain (31,32,34) (in *Aquifex aeolicus* the DnaA residues responsible for the interaction were determined by crystallography (32) and in *E. coli* DnaA by mutagenesis (34)). In this interaction two pairs of helices, α3/α4 and α5/α6, of ATP binding domain are involved, which geometry creates a single conduit along the length of DnaA filament (32). Each protomer of DnaA protein binds three nucleotides of ssDNA through van der Waal and salt-bridge interactions (32). Plasmids’ and archaeal OBPs possess a Winged Helix (WH) domain that is structurally different from the DBD of bacterial OBPs and specifically interacts with origin dsDNA. The WH structural motif is also typical of eukaryotic replication initiators. In plasmid Rep proteins, there are two such domains that bind directly to the repeated sequences (iterons) located adjacent to the DUE, resulting in the local destabilization of the duplex at the AT-rich region (30). To date no AAA+ domains have been identified in the structure of Rep proteins.

The nature of the specific nucleoprotein complex formed by bacterial DnaA proteins within the DUE has recently been investigated (31,32,34,38). The ability of replication initiators, which contain WH domains and no AAA+ domain, to form a complex with ssDNA in the AT-rich region has not been previously characterized. In this work, we show that plasmid OBPs containing WH domains can specifically interact with ssDNA within the DUE and that this interaction is critical for the initiation of DNA replication.

## MATERIALS AND METHODS

### Bacterial strains and plasmids

The following *E. coli* strains were used in this study: C600, CC118, CC118 (λpir) and BL21(DE3) (8). For purification of the TrfA-33 monomeric mutant (G254D/S267L), the pAT30 plasmid (39) was used. RepE R118P protein was purified from the pBK815 plasmid (40). Plasmids pKD19L1, pKD19L1 1–6 (7) and pZZ38 (41) were used in gel filtration and *in vitro* replication assays, and for transformation frequency determination. These plasmids were also used for preparation of electrophoretic mobility shift assay (EMSA) probes and of dsDNA fragments for the atomic force microscopy (AFM).

### Protein purification

Highly purified proteins (95% purity or higher) were utilized in experiments described in this study. Replication-active monomeric forms of TrfA-33 (TrfA-33 G254D/S267L) and RepE (RepE R118P) proteins were purified as previously described (39,42).

### Preparation of DNA probes and EMSA

DNA probes were prepared by DNA labeling with Alexa555-dCTP (Invitrogen) and Terminal deoxynucleotideyl Transferase (Promega). DNA labeling and purification were performed as previously described for DNA fragments labeled with Cy3-dCTP (43,44). dsDNA fragments, containing minimal origin region (consisting of the AT-rich region, the iterons and the DnaA-boxes), were prepared in polymerase chain reaction (PCR) reactions with primers oriV1 and oriV2 or oriS1 and oriS2 (see Supplementary Table S1 for oligonulecotide sequences). ssDNA oligonucleotides were commercially synthesized (Thermo Scientific) (Supplementary Table S1).

Reaction mixtures containing 1 pmol of the fluorescent DNA probe and increasing amount of Rep protein (5, 10, 15, 20 pmols of TrfA-33 G245D/S267L or of RepE R118P), in a buffer containing 40 mM HEPES-KOH pH 8, 25 mM Tris-HCl pH 7.6, 100 mM NaCl, 4% (w/v) sucrose, 4 mM dithiothreitol, 80 μg/ml BSA, were incubated for 20 min at 32ºC. After incubation 2.5% (v/v) Ficoll 4000 was added and reactions were loaded onto a 5% polyacrylamide gel. Gels were prepared in Tris-borate/EDTA buffer and, after electrophoresis, scanned with a Pharos FX Imager (Biorad).

### SPR analysis

Standard surface plasmon resonance (SPR) analyses using a BIAcore 2000 were performed essentially as described in the manufacturer's manual. DNA binding by TrfA-33 254D/267L was studied using a biotinylated dsDNA fragment containing five RK2 iterons or ssDNA oligonucleotides containing the sequence of the top or bottom strand of the RK2 DUE immobilized on a streptavidin matrix-coated Sensor Chip SA. As negative controls dsDNA fragments containing RK2 DUE*_oriV_* sequence and fragment of plasmid pUC18 were used. All oligonucleotides were commercially synthesized (Thermo Scientific) (Supplementary Table S1). Running buffer was HBS-EP (150 mM NaCl, 10 mM HEPES pH 7.4, 3 mM EDTA, 0.005% Surfactant P20). In all experiments the buffer flow was set to 15 μl/min with all injections at a volume of 30 μl. The results are presented as sensorgrams obtained after subtraction of the background response signal from control experiments with buffer injections.

### Gel filtration assay

To analyze the formation of a tripartite nucleoprotein complex, a column gel filtration method with Sepharose CL-4B was used. Reaction mixtures (120 μl) containing 190 nM TrfA G254D/S267L or RepE R118P protein and 2.5 nM of supercoiled plasmid DNA containing minimal origin region, pKD19L1 1–6 or pZZ38, in reaction buffer (40 mM HEPES-KOH pH 7.6, 25 mM Tris-HCl, pH 7.6, 4% (w/v) sucrose, 4 mM dithiothreitol, 80 μg/ml BSA) were prepared. The mixtures were incubated for 15 min at 32ºC, then the appropriate ssDNA oligonucleotides (17 nM), fluorescently labeled with Alexa555 dye, were added and mixtures were further incubated for 15 min. Then the mixtures were applied on the CL-4B column (0.5 × 12 cm) and run in column buffer (40 mM HEPES-KOH pH 7.6, 40 mM potassium glutamate, 4% (w/v) sucrose, 4 mM dithiothreitol, 10 mM magnesium acetate, 0.01% Brij-58). Two-drop (80 μl) fractions were collected and analyzed for detection of fluorescently labeled ssDNA with a DTX880 Multimode Reader. Ten microliter samples from fractions were run on an agarose gel with ethidium bromide to visualize DNA.

### AFM

DNA fragments containing the origin regions (including the AT-rich region, the iterons and the DnaA-boxes) of RK2 and F plasmids for AFM imaging were prepared by restriction digestion or PCR. Restriction digestion of pKD19L1 with *Pst*I and pZZ38 with *Eco*RI and *Hin*dIII restriction enzymes resulted in dsDNA fragments 1615 bp and 1132 bp, respectively. Shorter DNA fragments, 435 bp containing the RK2 plasmid origin region and 217 bp containing the F plasmid origin region, were obtained by PCR reactions with primers oriV1 and oriV2 and oriS1 and oriS2 (Supplementary Table S1).

For imaging nucleoprotein complexes with AFM, 20 μl reaction mixtures of 225 nM Rep protein (either TrfA-33 G254D/S267L or RepE R118P), and the appropriate dsDNA fragments were incubated in a buffer containing 25 mM Tris-HCl, pH 7.6, 11 mM MgCl_2_ and 4 mM dithiothreitol. To prevent protein aggregation at 200 nM, 100 mM NaCl was also included in some experiments. Mixtures were incubated at 32ºC for a maximum of 10 min. Next, the ssDNA oligonucleotides containing the sequence of the top or bottom strand of the DUE were added to the reaction mixture, incubated at 32ºC for up to 10 min and placed onto a freshly cleaved mica surface. After 30 s, the mica surface was washed with filtered-MilliQ water (Millipore, Billerica, MA) and blown dry in a gentle stream of nitrogen gas.

Samples were imaged in air at room temperature and low humidity using tapping mode with amplitudes of 5 nm and scan rates of 2 lines·s^−1^ on an AFM from Nanotec (Nanotec Electrónica, Madrid, Spain) with PointProbePlus type PPP-NCH tips (Nanosensors, Neuchâtel, Switzerland). Standard image processing consisted of plane subtraction and flattening using WSxM freeware (45). The color scale in all AFM images (from dark to white) is 0–2.5 nm.

### Transformation frequency

Plasmid DNA was purified through two CsCl buoyant density gradients and 100 ng was used for transformations. Cells were prepared for electroporation as described previously (8,46). Electroporation was performed in 2 mm cuvettes (Bio-Rad) and the parameters were 3000 V, 25 μF, 200 Ω. The transformation frequency was calculated as colony forming unit (CFU) per 1 μg of plasmid DNA.

### *In vitro* replication in crude extract

Reactions were performed as described previously (47) using *E. coli* C600 crude extract with plasmid DNA templates (300 ng) and TrfA-33 G254D/S267L (200 ng). The construction of DNA templates containing a mutated *oriV* region was done as described by Kowalczyk *et al.* (8) with the use of the following oligonucleotides: swap R top, swap R bottom, swap M2-R top, swap M2-R bottom, swap M1-R top and swap M1-R bottom (Supplementary Table S1).

## RESULTS

### Plasmid Rep proteins bind a specific single strand of the DUE DNA in the origin region

**Figure 2.**
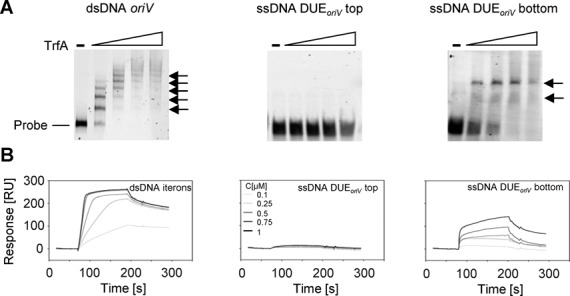
TrfA protein binds one specific strand of ssDNA in the DUE*_oriV_* of the origin. The interaction of TrfA and DNA was analyzed with an EMSA (A) and SPR (B) as described in Materials and Methods. (**A**) Increasing amounts of TrfA protein (5, 10, 15, 20 pmol) were incubated with 1 pmol fluorescently labeled dsDNA containing *oriV*, ssDNA of DUE*_oriV_* top strand or ssDNA of DUE*_oriV_* bottom strand as noted. Black arrows indicate nucleoprotein complexes. (**B**) Increasing amounts of protein (0.1, 0.25, 0.5, 0.75, 1 μM) were run over the surface of a sensor chip with immobilized dsDNA containing iterons or ssDNA of DUE*_oriv_* the origin region, top or bottom strand, as indicated.

Previous investigations of nucleoprotein complexes formed in the DUE did not include Rep proteins, which contain WH domains and no AAA+ domain. To determine if this class of OBP behaved similar to DnaA, we analyzed complex formation with ssDNA DUE and the plasmid replication initiation proteins, TrfA from plasmid RK2 and RepE encoded on plasmid F. In all experiments mutants of the initiator proteins were used, TrfA-33 G254D/S267L or RepE R118P, which are constantly monomers, active during replication initiation. The use of monomeric mutants eliminated the necessity of activation of the replication-inactive Rep dimers by chaperones to replication-active monomers. Using gel retardation assays, we studied the ability of TrfA to bind to ssDNA oligonucleotides containing the sequence of the top or bottom strand DUE of *oriV* (ssDNA DUE*_oriV_*). When TrfA was incubated with the bottom strand ssDNA, we observed two bands migrating slower than the free probe (Figure 2A, middle and right panels). Such retarded bands were not detected when top strand ssDNA was used. As a control, we analyzed the complexes formed between TrfA protein and dsDNA containing the minimal *oriV* region. This resulted, as expected, in five retarded bands corresponding to TrfA-iterons complexes (Figure 2A, left panel). The interaction between TrfA and ssDNA was also analyzed with SPR (Figure 2B). In agreement with gel-shift data, a TrfA-ssDNA complex was only observed when oligonucleotides containing the sequence of the bottom strand of DUE*_oriV_* were immobilized on the sensorchip surface. When oligonucleotides comprising the top strand sequence were used, an interaction between the TrfA protein and the ssDNA was not detected. Moreover, consistent with the intensity of bands detected in the EMSA, we confirmed that TrfA binds the dsDNA iterons of *oriV* with a higher affinity then the bottom strand ssDNA. Control experiments with dsDNA containing the sequence of DUE region and a dsDNA fragment of pUC18 plasmid showed only slight interaction of TrfA protein with the DNA sequence lacking specific binding motifs (Supplementary Figure S1). These interactions were comparable with TrfA binding to ssDNA DUE*_oriV_* top and were much weaker than with dsDNA iterons and ssDNA DUE*_oriV_* bottom.

Similar results were observed for another replication initiator, the RepE protein from plasmid F (Supplementary Figure S2). This protein also formed complexes with dsDNA containing iterons from the F plasmid *oriS* region as well as with ssDNA containing the sequence of only one of the strands of the DUE of *oriS* (ssDNA DUE*_oriS_*).

### Plasmid Rep proteins bind iteron dsDNA and DUE ssDNA simultaneously

**Figure 3.**
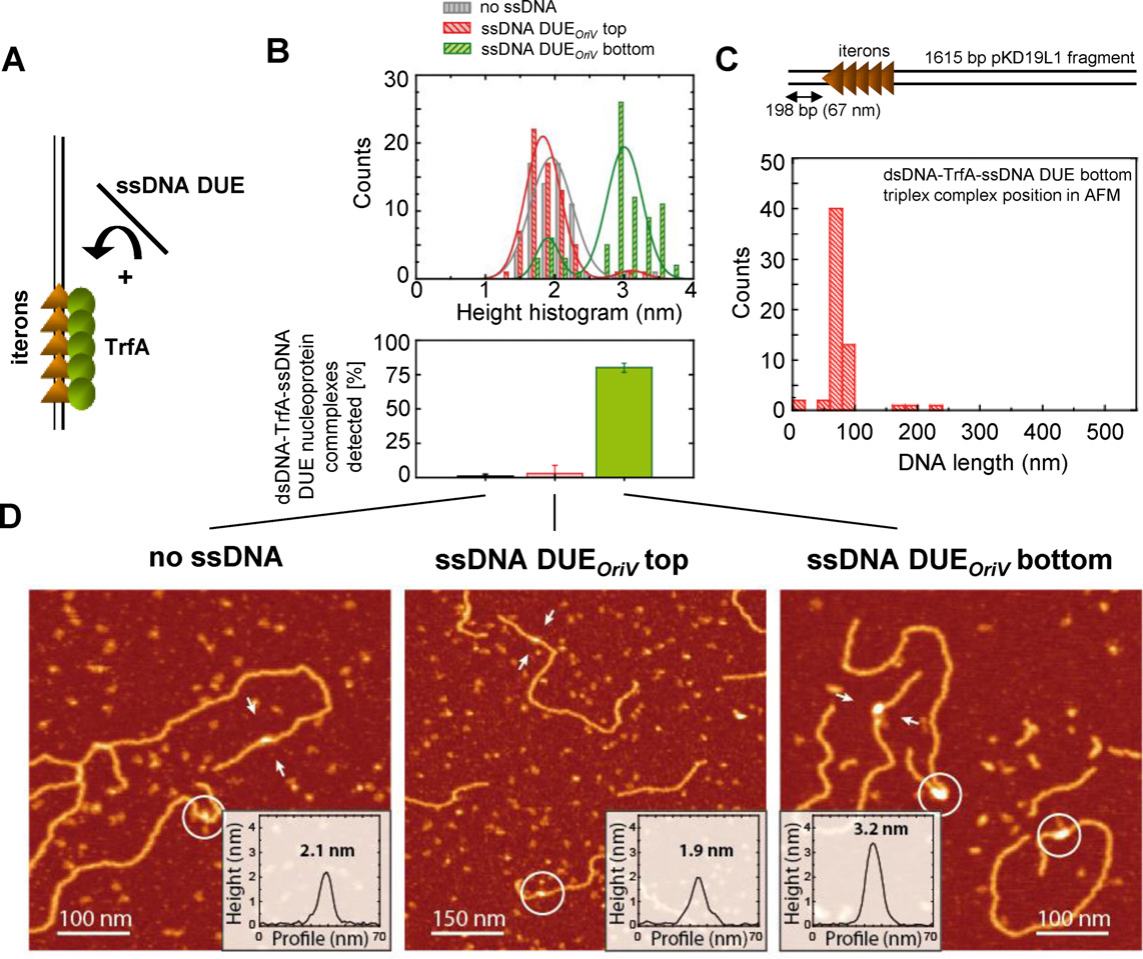
AFM characterisation of TrfA dipartite and tripartite complexes. (**A**) Scheme of the experiments: TrfA protein was first incubated with linear dsDNA containing iterons and then ssDNA containing the sequence of the top or bottom strand of DUE*_oriV_* was added. (**B**) Top panel: height histogram of TrfA dipartite and tripartite complexes: dsDNA-TrfA (no ssDNA), dsDNA-TrfA-ssDNA top (ssDNA DUE*_oriV_* top) and dsDNA-TrfA-ssDNA bottom (ssDNA DUE*_oriV_* bottom); bottom panel: occurrence of tripartite complexes. (**C**) Top panel: depiction of the iterons’ position on the dsDNA substrate; bottom panel, histogram showing the binding position of tripartite complexes on the dsDNA substrate. (**D**) Typical AFM images of experiments. Characteristic profiles enclosed with white arrows are shown as insets. Other examples of complexes are encircled. Colour scale in AFM images (from dark to white) is 0–2.5 nm.

The binding of Rep proteins to iteron sequences results in the local melting of dsDNA and the formation of a replication bubble consisting of ssDNA in the origin region. Since our EMSA and SPR experiments demonstrated that Rep proteins could bind to the dsDNA containing iterons and to the ssDNA containing a sequence of just one specific strand of the DUE, we wanted to determine if the Rep proteins could form a complex with both dsDNA and ssDNA simultaneously. To answer this question we employed AFM and gel filtration assays. We first used a linear dsDNA fragment (1615 bp) with the iteron sequences starting 198 bp from one end. The dsDNA fragments were first incubated with TrfA and then with ssDNA of DUE*_oriV_*, top or bottom strand (Figure 3). AFM provides a topographic map of the surface of molecules and thereby allows identification of protein–DNA interactions from height measurements (Supplementary Figure S3). Bare dsDNA showed a mean height around 1 nm in agreement with published results (48). TrfA–DNA interactions showed a mean height of 1.9 ± 0.2 nm (see histogram of heights in Figure 3B, grey data, *N* = 66 and Supplementary Figure S3A). We detected the formation of a tripartite complex between TrfA and both dsDNA and ssDNA when the ssDNA contained the sequence of the bottom strand of DUE*_oriV_*. These tripartite nucleoprotein complexes exhibited a mean height of 3 ± 0.2 nm (see Figure 3B, green data, *N* = 78 and Supplementary Figure S3A). The tripartite complexes were observed in approximately 80% of the molecules examined (Figure 3B, bottom panel), and occurred precisely at the iteron region (Figure 3C). Experiments where ssDNA containing the sequence of the top strand of DUE*_oriV_* was used showed a mean height of 1.9 ± 0.2 nm (see Figure 3B, red data, *N* = 68), similar to the control experiment with no ssDNA added to the sample. The number of tripartite complexes observed in this experiments was almost as low as the number observed in the control with no ssDNA added to the sample (Figure 3B, bottom panel). Insets in Figure 3D and Supplementary Figure S3 show examples of height profiles. Molecules of tripartite complex height (∼3 nm) were not detected in control experiments where TrfA, dsDNA and ssDNAs were incubated separately or in mixtures containing only two of the three components (Supplementary Figure S4). The formation of tripartite complexes between dsDNA, TrfA and the bottom strand of the ssDNA DUE*_oriV_* was also observed in experiments utilizing a shorter dsDNA iteron fragment (435 bp) (Supplementary Figure S5A). Previous work reported a shortening of DNA upon DnaA binding (49). Motivated by that finding we imaged Rep–DNA complexes using these shorter DNA molecules. However, our measurements could not reach a reliable conclusion regarding shortening considering the distance resolution of our AFM setup in this experiment (about 40 bp).

**Figure 4.**
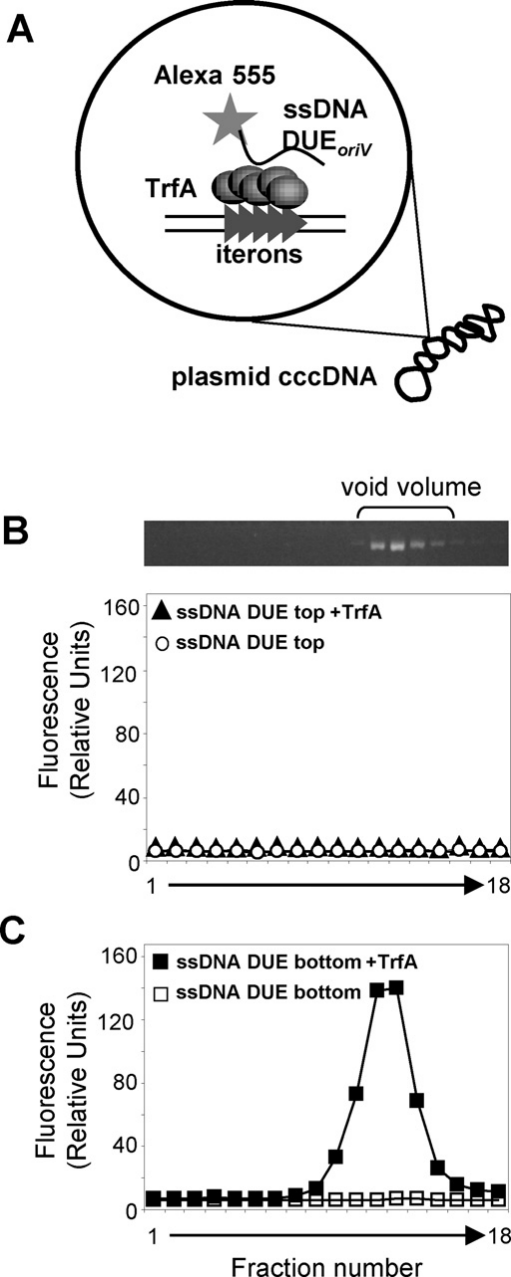
TrfA protein forms a complex consisting of plasmid cccDNA and ssDNA of the DUE*_oriV_* bottom strand. (**A**) The scheme of experiment. The supercoiled plasmid DNA containing iterons was incubated with TrfA protein and then fluorescently labeled ssDNA containing the DUE*_oriV_* sequence of the top (**B**) or bottom (**C**) strand was added (solid symbols). In control experiments, no TrfA protein was added (open symbols). After incubation the mixture was separated using size exclusion chromatography. Fractions were analyzed for the presence of plasmid cccDNA (top of the panel B) and fluorescently labeled ssDNA (graphs). Co-localization of the ssDNA and plasmid DNA in the void volume fractions indicates tripartite complex formation.

Similar results to those obtained with the linear DNA fragments were observed in gel filtration assay with supercoiled plasmid DNA and fluorescently labeled ssDNA probes. The plasmid used in these experiments, pKD19L1 1–6, contains a mutation within the origin region that prevents melting of the dsDNA when TrfA binds to the iterons. Therefore, the only ssDNA present in the reaction mixture was that of the additionally added fluorescently labeled one. The incubation of TrfA, pKD19L1 1–6 and fluorescently labeled ssDNA followed by size exclusion separation of the nucleoprotein complexes formed (Figure 4A) resulted in the detection of fluorescent signal in those fractions containing supercoiled plasmid DNA only when the reaction mixture included the ssDNA of the DUE*_oriV_* bottom strand (Figure 4C). The detection of a fluorescent signal from the ssDNA DUE*_oriV_* bottom strand and supercoiled plasmid DNA in the same fractions indicated the formation of a tripartite nucleoprotein complex. The tripartite complex was not detected when ssDNA containing the sequence of the top strand of DUE*_oriV_* was used (Figure 4B) or when there was no TrfA protein added to the reaction mixture (Figure 4B and C).

The observed formation of a tripartite complex between dsDNA containing iterons, the plasmid replication initiator protein and ssDNA of just one strand of DUE was not restricted to the TrfA replication initiation protein. Similar results were obtained when the RepE protein encoded by F plasmid was used (Supplementary Figures S5–S7) with the appropriate DNA elements from the F replication origin. RepE was capable of tripartite complex formation when ssDNA of the top strand of DUE*_oriS_* from F plasmid was incubated with the protein and dsDNA containing the *oriS* region, either linear (217 bp and 1132 bp) (Supplementary Figures S5B and S6) or supercoiled (Supplementary Figure S7). Such nucleoprotein complexes were not detected when no ssDNA was added or when ssDNA containing the sequence of the bottom strand of DUE*_oriS_* was added to the mixture of dsDNA and RepE protein. In the latter case, only a few molecules (approximately 20%) formed a tripartite complex. The tripartite complex of dsDNA-RepE-ssDNA DUE*_oriS_* top strand had the expected characteristic height while scanning with AFM (Supplementary Figure S3B) and its position within the dsDNA fragment corresponded to the location of the iterons (Supplementary Figure S6C). Structures with such height were not observed in control experiments (Supplementary Figure S8).

### All four 13-mers within the DUE_oriV_ are required for binding by TrfA

**Figure 5.**
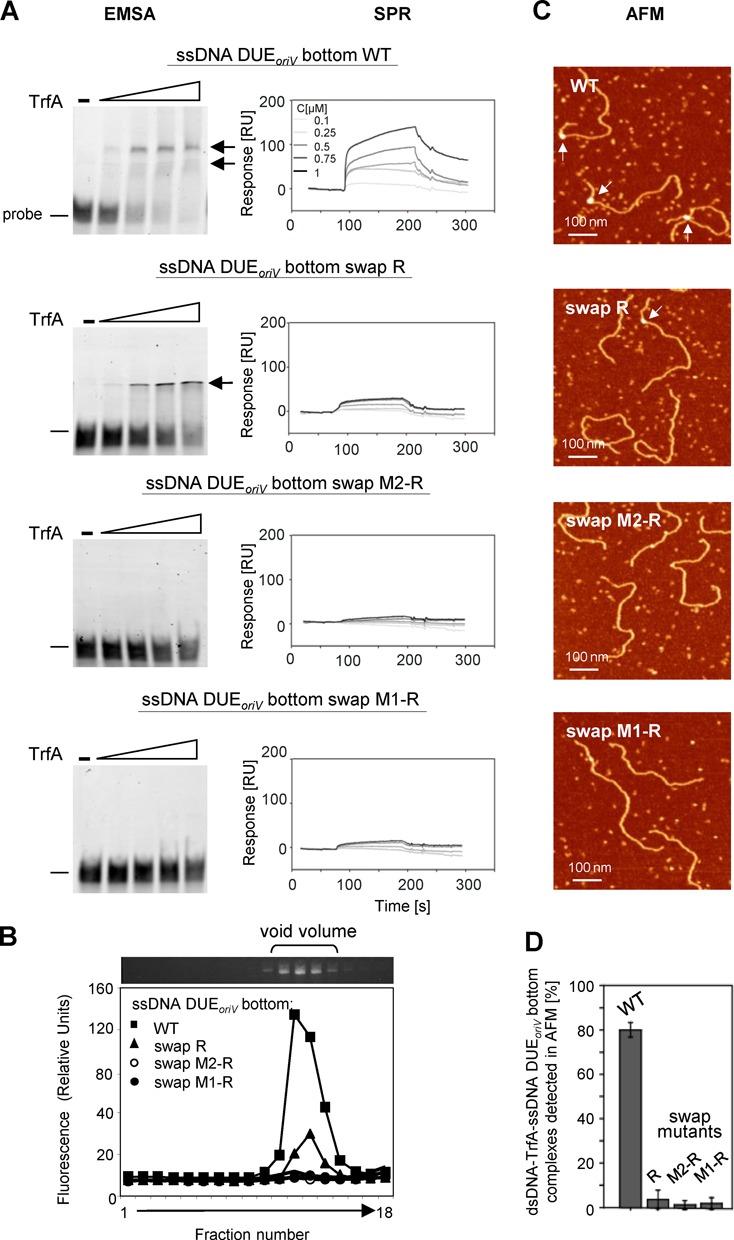
Formation of a tripartite nucleoprotein complex at *oriV* requires all four 13-mers of the DUE*_oriV_* bottom strand. ssDNA DUE*_oriV_* sequence requirements for binding by the TrfA protein were tested with an EMSA and SPR (**A**). TrfA interaction with wild type and mutated oligonucleotides of the DUE*_oriV_* bottom strand were analyzed. Increasing amounts of TrfA (5, 10, 15, 20 pmol) were used in the EMSA. Arrows indicate detected nucleoprotein complexes. For SPR analysis 0.1, 0.25, 0.5, 0.75 and 1 μM TrfA concentrations were used. For analysis of the sequence requirements for tripartite nucleoprotein complex formation with plasmid cccDNA and ssDNA, size exclusion chromatography, as detailed in Materials and Methods, was used (**B**). For sequence requirements of tripartite nucleoprotein complex formation with linear dsDNA and ssDNA, AFM analysis (see Materials and Methods) was used (**C, D**). White arrows on the panel C images indicate tripartite complexes.

There are four 13-nucleotides repeats within the DUE of the plasmid RK2 origin: left (L), middle 1 (M1), middle 2 (M2) and right (R) (Figure 1). The EMSA, SPR, AFM and gel filtration analysis clearly showed that the TrfA protein binds just one strand of the DUE (Figures 2–4). However, the question remained whether all four 13-mers were required for nucleoprotein complex formation. To answer this question we used ssDNA 70-nucleotide oligonucleotides containing all four (wild type), three (swap R), two (swap M2-R) or just one (swap M1-R) of the 13-mers of the DUE*_oriV_* bottom strand. These oligonucleotides contained, apart from the sequence of the particular 13-mers of the bottom strand, the sequence of the top strand of DUE*_oriV_* for the deleted sequences. These ssDNA oligonucleotides were then used in EMSA and SPR analysis (Figure 5A). Both techniques showed that changes in the sequence of even one of the 13-mers (ssDNA DUE*_oriV_* bottom swap R) reduced the binding of TrfA when compared to the wild-type ssDNA. In the EMSA experiments with the ssDNA of the DUE*_oriV_* bottom swap R only one retarded band was observed instead of the two bands visible when wild-type ssDNA of the DUE*_oriV_* bottom strand was used (Figure 5A). The relative response obtained during the SPR analysis with the swap *R* oligonucleotide immobilized on sensor chip and injections of TrfA was over five times lower in comparison to the analysis performed using the wild-type sequence oligonucleotide (Figure 5A). When oligonucleotides containing the sequence of two 13-mers (ssDNA DUE*_oriV_* bottom swap M2-R) or just one 13-mer (ssDNA DUE*_oriV_* bottom swap M1-R) of the bottom strand were used, no interaction between TrfA and the ssDNA was observed using either technique.

The same oligonucleotides were also used to analyze the tripartite complex formed between TrfA protein, ssDNA (wild type or mutant sequences) and either linear dsDNA (Figure 5C and D; analysis using AFM) or supercoiled DNA (Figure 5B; analysis using gel filtration). Both techniques showed that TrfA bound dsDNA and ssDNA simultaneously only when the ssDNA contained the sequence of all 13-mers of the DUE*_oriV_* bottom strand. Swapping the sequence of even one 13-mer between the strands resulted in a reduction in tripartite nucleoprotein complex formation. Quantification of AFM images showed a drastic reduction of tripartite complex formation occurrence from 80% to 10% (or less) when mutated oligonucleotides were used (Figure 5D). In addition, in the gel filtration assay a significant reduction of fluorescent signal coming from labeled ssDNA bound in a tripartite nucleoprotein complex was observed when the swap mutants were utilized (Figure 5B).

### Sequence of all 13-mers within the ssDNA DUE_oriV_ is required for origin replication activity

Changes within the sequence of the DUE bottom strand reduced the ability of TrfA to interact with the ssDNA. To check if disturbances in this interaction would influence plasmid replication, we performed *in vitro* replication experiments using crude cellular extract of *E. coli* C600 as well as transformation frequency tests for *in vivo* activity. In these assays pKD19L1 plasmid derivatives containing an altered DUE *oriV* region were used. In these derivatives, the bottom strand of the DUE*_oriV_* contained the sequence of three (L, M1 and M2), two (L and M1) or just one (L) 13-mer out of four that are present in the wild-type sequence. The rest of the sequence of the DUE*_oriV_* bottom strand was swapped with the sequence of DUE*_oriV_* top strand, which resulted in three mutants: swap R, swap M2-R and swap M1-R. The replication assays in crude extract revealed that none of the tested mutants retained replication activity *in vitro*. Also, no activity was observed when TrfA protein was omitted from the reaction mixture (data not shown). The altered plasmids were also inactive *in vivo* in *E. coli* CC118 strain. No colonies were obtained on agar plates, when the plasmids’ replication relied on the mutated *oriV* region and TrfA protein (Table 1). In control experiments, bacterial strain *E. coli* CC118 (λpir), which encodes π protein on its chromosome, was utilized. Since the pKD19L1 plasmid derivatives used in our experiments possess an additional origin (*oriγ*) from R6K plasmid, in the presence of the π protein their replication relies on *oriγ*. This *oriV*-independent replication activity allowed us to test the quality of the altered plasmid DNA in transformation tests. All tested pKD19L1 variants were active in this control (Table 1).

**Table 1.** *In vitro* and *in vivo* replication activity of *oriV* with altered DUE bottom strand sequence

	Replication activity *in vitro*		Transformation frequency	
*oriV* template	pmol	%	CC118 (CFU μg-1 DNA)	CC118 (λpir) (CFU μg-1 DNA)
WT	90 ± 10	100	8 × 10^6^	8.5 × 10^6^
swap M1-R	2.0 ± 0.05	2.2	0	3 × 10^6^
swap M2-R	2.1 ± 0.2	2.3	0	6.3 × 10^6^
swap R	1.7 ± 0.3	1.9	0	1.5 × 10^6^
Activityof origin mutants in *in vitro* replication assaywas tested in reaction mixtures containing *E. coli* C600 cellular extract(fraction II), plasmid cccDNA and monomeric TrfA protein. The assay wasperformed under standard replication conditions (8). Total nucleotide incorporation (pmol) and relativereplication activity (%) are reported. The value obtained for the wild-type DNAtemplate was taken as 100% and the replication activity of altered templateswas normalized to the wild-type template. *In vivo* the activity of thealtered *oriV* templates was analyzed by the transformation assay (seeMaterials and Methods) in *E. coli* CC118 strain and as a control in *E.coli* CC118 (λpir). Transformation frequency is reported as CFUper 1 μg of plasmid DNA used for transformation of bacteria.				

## DISCUSSION

**Figure 6.**
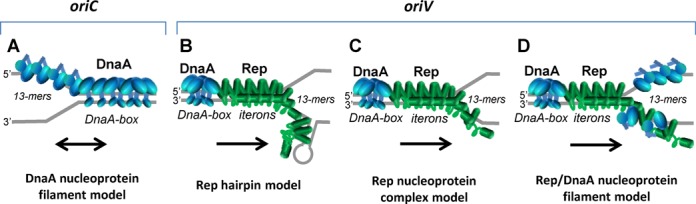
Alternative models of nucleoprotein complex formation at the *origin* region by bacterial and plasmid replication initiators. In the presented models the regions of *oriC* and *oriV* origins, and initiator proteins, DnaA (blue) and TrfA (green) are marked. (**A**) Model of binding of the DnaA protein (blue) to dsDNA containing DnaA-boxes and to ssDNA DUE containing three 13-mers. DnaA-ATP forms a filament at the DnaA-boxes. Such filament extends to the 13-mers at the ssDNA. The model, based on Duderstadt *et al.* (31), presents only the AAA+ and DBD domains of DnaA. (**B–C**) Alternative models of binding of the plasmid RK2 replication initiator TrfA protein at the dsDNA containing iterons and the ssDNA DUE containing the four 13-mers. (**B**) TrfA protein binding to the iterons results in DUE melting. The melted ssDNA forms local secondary structures that are further stabilized by the TrfA. This process is accompanied by DnaA protein bound to four DnaA-boxes. (**C**) TrfA protein binds to iterons which results in DUE melting. Then, it extends along one of the two single strands of DNA. The DnaA protein bound to four DnaA-boxes is shown in blue. (**D**) The coordinated binding of TrfA protein to iterons and the DnaA protein to DnaA-boxes causes melting of the DUE. Then the TrfA and DnaA could bind to the same DNA strand forming a heterofilament or each of the proteins could bind to different strands. The orientation of the origin models is as presented by Duderstadt *et al.* (31) (A) and Kowalczyk *et al.* (8) (B–D). Black arrows in the illustrations indicate the direction of replication. The proposed models do not exclude discontinuity of Rep protein filaments, neither aim to state the nature or structure of tripartite complexes (see the discussion for details).

To date all biochemical and structural data on the formation of nucleoprotein complexes of replication initiators at the ssDNA DUE region of the replication origin have come from studies with bacterial DnaA proteins, which possess a DBD and an AAA+ domain (Figure 6A; model of DnaA nucleoprotein complex). In this work, we show that the replication initiators from plasmids RK2 and F that possess WH and lack ATP binding domain can also form a nucleoprotein complex at the DUE and that the complex is specific to one of the two single strands. Furthermore, we show that all repeated sequences within the DUE of plasmid RK2 are indispensable for stable binding by the plasmid replication initiator and for replication activity of the plasmid origin. Based on our data and on the knowledge gained from bacterial systems, we discuss three hypothetical models of nucleoprotein complex formation at plasmid DUE (Figure 6B–D).

### Rep hairpin model

Our results showed that both Rep proteins analyzed, TrfA and RepE, form complexes with only one strand of the DUE. In the EMSA experiments two retarded bands were observed, indicating the formation of two types or two steps of complex formation by both TrfA and RepE. This might indicate that both proteins form complexes within the ssDNA DUE in a similar manner. The analysis of the nucleoprotein complexes formed between TrfA and mutated ssDNAs of the DUE*_oriV_* bottom strand indicates that the precise sequence of all four 13-mers is needed for stable binding of the replication protein. In EMSA experiments with the ssDNA DUE*_oriV_* swap R mutant only one type of the complex, observed as one retarded band, was formed. In an experiment with the swap M2-R mutant there was no retardation at all. One possible explanation for those results could be the formation of secondary structures within the ssDNA DUE*_oriV_* bottom strand, which are bound by TrfA protein (Figure 6B). *In silico* analysis of possible secondary structure formation by the ssDNA DUE*_oriV_* (Supplementary Figure S9A and B) showed that the bottom strand of DUE*_oriV_* indeed could form long hairpin structure, which was not predicted for the top strand. For analyzed sequences of the mutated bottom strand (Supplementary Figure S9C–E) the secondary structure formation was also predicted, however, even for the swap R mutant the hairpin was not as long as the one modeled for the wild-type bottom strand. Perhaps, if the hairpin model is valid, the binding of plasmid Rep protein to the local secondary structure within the ssDNA DUE is efficient only for long hairpins. Moreover, the TrfA binding to the secondary structure within ssDNA DUE*_oriV_* bottom should be sequence specific because TrfA interacts weakly with dsDNA lacking specific binding motifs (e.g. dsDNA DUE region, dsDNA fragment of pUC18 plasmid). However, the discussed hairpin model is not supported by prediction of hairpin structure formation within the ssDNA DUE*_oriS_* (Supplementary Figure S9F and G) because hairpin structures have not been predicted for both top and bottom strand of *oriS*. It should be noted that the *in silico* analysis does not survey supercoiling of the plasmid DNA in the proximity of ssDNA DUE, which does take place in a bacterial cell.

### Rep nucleoprotein complex model

Interestingly, studies on DnaA proteins showed that these replication initiators, structurally different from plasmid Rep proteins, also bind specifically to just one strand of the ssDNA DUE of the chromosomal origin (32,34,50). Similar to our studies with TrfA, the analysis of the ability to bind mutated ssDNA DUE of *E. coli* chromosomal *oriC* by DnaA protein also showed that only the wild-type sequence is effectively bound by the replication initiator (34). These results indicate that the interaction with ssDNA in an origin region, where dsDNA melts, could be a common mechanism not only in bacterial chromosomes, but also in other replicons, such as plasmids. Since eukaryotic OBPs possess a WH domain similar to plasmid replication initiators, the knowledge gained from studies with the Rep proteins could possibly be exploited in studies of eukaryotic systems. In bacterial DnaA proteins, the AAA+ domain is crucial for the interaction with the ssDNA DUE (31,32,34). However, the plasmid Rep proteins do not contain AAA+ domain responsible for nucleotide binding. Therefore, it is possible that the WH domains, involved in binding of iterons within the dsDNA origin, also bind the ssDNA originating after dsDNA melting (Figure 6C). It is, however, not known whether binding of these two forms of DNA is combined with changes in conformation of Rep protein. For DnaA protein it was proposed that the dsDNA–DnaA complex is formed by a more extended DnaA form, exposing a helix-turn-helix motif for engaging the DnaA-boxes, whereas ssDNA DUE is bound by a more compact form of the protein (31) (Figure 6A). It was also shown, using crystallography, that the DnaA protein from *A. aeolicus* covering the ssDNA forms a helical filament (32). In the case of plasmid Rep proteins, such oligomeric structures have not been observed. Since we observed two discreet bands in EMSA, at least two monomers of Rep protein particles take part in the observed nucleoprotein complex. However, we cannot exclude that, similar to the DnaA protein, TrfA and RepE proteins might also form a helical filament on ssDNA. It seems possible when we consider the hydrophobic structure of the Rep proteins that makes them susceptible to the formation of higher oligomeric structures.

Although the exact structure of Rep proteins bound to dsDNA and ssDNA and their mechanism to form nucleoprotein complexes is unknown, our AFM observations and gel filtration assays showed that the Rep proteins possess the ability to interact with the dsDNA containing iterons and ssDNA with DUE sequence simultaneously. However, our data could not determine beyond all doubts if single Rep proteins bind at the same time to dsDNA and ssDNA or if tripartite complexes are formed through protein–protein interactions of Rep molecules binding either to ssDNA or dsDNA. Undoubtedly, tripartite nucleoprotein complexes were formed only with ssDNA composed of the sequence of one strand of the DUE containing all repeated sequences. The deletion of just one of the repeats resulted in a decrease in the number of tripartite complexes detected. The same deletion resulted in the inactivation of the plasmid origin in both *in vitro* and *in vivo* replication. It could be suspected that binding of ssDNA DUE by Rep protein stabilizes the formation of the open complex and that introducing changes in the DUE sequence abolishes opening. It can not be excluded that the changes in DUE sequence not only disturb the binding of Rep protein but also influence other steps of the replication initiation, what may result in inactivation of replication (e.g. action of host DnaA protein, helicase complex or replisome assembling). Indeed, specific mutations in DUE have been shown to abolish helicase complex activity but not origin opening (8,9) nor the TrfA interaction with ssDNA DUE (our unpublished results). The binding of replication initiator protein to dsDNA resulting in DUE melting and then binding of initiator to ssDNA DUE seem to be the very first steps of replication initiation, and when abolished they are limiting the whole process.

In our experiments analyzing the tripartite complex formed by Rep proteins, we used dsDNA molecules that could not be opened either because they were linear, or they were mutated to prevent opening (pKD19L1 1–6), or the assays did not include DnaA protein which assists in the melting of the AT-rich region of the F origin (51,52). All conditions resulted in nucleoprotein formation composed of Rep proteins interacting with iterons and specific ssDNA of the DUE. Similar conditions using linear dsDNA and ssDNA fragments were used in experiments with *E. coli* DnaA protein. These results showed that the bacterial initiator binds simultaneously to both DnaA-boxes within the double-stranded region of the origin and the ssDNA (34,38,53). The efficient formation of a tripartite complex between the bacterial initiator, dsDNA origin and ssDNA of the DUE required oligomerization of DnaA protein on the DUE-flanking region (DnaA-box R1, Integration Host Factor binding site) and low-affinity binding sites (DnaA-box R5, I1, I2, τ1, τ2). However, the high-affinity DnaA-box R1, nearest to the AT-rich region, is sufficient for the DnaA-ssDNA interaction (38). These data seem to contradict the continuous DnaA filament model introduced by Duderstadt *et al.* (31), however, they do not exclude it. The formation of tripartite nucleoprotein complex by the DnaA protein (38), the necessity of strict spacing between the DnaA-boxes and DUE (38,54) and DnaA oligomers assembly occurring in the absence of any high-affinity DnaA-boxes (55) suggest that the filament of DnaA protein may not be continuous. The proposed model was called the ssDUE recruitment model (38). The formation of tripartite nucleoprotein complex by TrfA and the importance of the exact spacing between iterons and DUE (7) suggest that this ssDUE recruitment model is also possible in case of plasmid replication initiators.

### Rep/DnaA nucleoprotein filament model

Replication initiation of some plasmids requires both the bacterial host DnaA protein in addition to the plasmid-encoded Rep protein (14,51,52,56–58). Such a situation occurs in the case of both RK2 (57,59) and F (51,52) when they replicate in *E. coli* cells. We cannot exclude the possibility that the DnaA protein either forms filaments on strands opposite to the one bound by Rep protein or that both Rep and DnaA proteins form hetero-nucleoprotein complexes on the same strand of ssDNA DUE (Figure 6D). However, all our experiments showed that Rep proteins alone are sufficient for complex formation with ssDNA DUE at the plasmid origin and that the DnaA protein was not required for this nucleoprotein structure. Additionally, DnaA was not necessary for the formation of Rep protein tripartite complex with dsDNA and ssDNA DUE. Although we found that *E. coli* DnaA protein can bind ssDNA DUE, both top and bottom strands of the RK2 plasmid *oriV*, we did not observe a tripartite complex formed by DnaA, plasmid cccDNA and ssDNA DUE (unpublished results). Therefore, the formation of the DnaA filament on plasmid ssDNA DUE or Rep–DnaA nucleoprotein complex seems to be improbable. However, we cannot exclude the possibility that the host initiator somehow cooperates with the Rep protein in nucleoprotein complex formation in DUE. This hypothesis requires future investigation.

## SUPPLEMENTARY DATA

Supplementary Data are available at NAR Online.

SUPPLEMENTARY DATA
