# Supplementary material for: Sequence-specific interactions of Rep proteins with ssDNA in the AT-rich region of the plasmid replication origin
Source: Nucleic Acids Res. 2014 May 16;42(12):7807–18. doi: 10.1093/nar/gku453 (PMC4081077; doi:10.1093/nar/gku453)
Supplement: SUPPLEMENTARY DATA [file supp_42_12_7807__index.html]

Sequence-specific interactions of Rep proteins with ssDNA in the AT-rich region of the plasmid replication origin — Sequence-specific interactions of Rep proteins with ssDNA in the AT-rich region of the plasmid replication origin — Sequence-specific interactions of Rep proteins with ssDNA in the AT-rich region of the plasmid replication origin — SUPPLEMENTARY DATA 

# Sequence-specific interactions of Rep proteins with ssDNA in the AT-rich region of the plasmid replication origin

## SUPPLEMENTARY DATA

**Files in this Data Supplement:**

- SUPPLEMENTARY DATA
