# Supplementary material for: Sequence-specific interactions of Rep proteins with ssDNA in the AT-rich region of the plasmid replication origin
Source: Nucleic Acids Res. 2014 May 16;42(12):7807–18. doi: 10.1093/nar/gku453 (PMC4081077; doi:10.1093/nar/gku453)
Supplement: SUPPLEMENTARY DATA [file supp_gku453_nar-03511-v-2013-File009.pdf]

oligonucleotides for	sequence
**replication**	
swap M1-R top	5'GATCTGGGTACCGCATTGGACAGAAAAAACCTGCTTTTAA ACCAATATTTATAAACCTTGTTTTTAACCAGGGCTGCGC3'
swap M1-R bottom	5'TCAGCGCAGCCCTGGTTAAAAACAAGGTTTATAAATATTGGTTTAAAA GCAGGT TTTTTCTGTCCAATGCGGTACCCA3'
swap M2-R top	5'GATCTGGGTACCGCATTGGACAGAAAAATTGGACGAAAAAAACCA ATATTTATAAACCTTGTTTTTAACCAG GGCTGCGC3'
swap M2-R bottom	5'TCAGCGCAGC CCTGGTTAAAAACAAGGTTTATAAATATTGGTT TTTTTCGTCCAATTTTTCTGTCCAATCGGTACCCA3'
swap R top	5'GATCTGGGTACCGCATTGGACAGAAAAATTGGACGAAAATTTGGTTATA AATTAAACCTTGTTTTTAACCAGGGCTGCGC3'
swap R bottom	5'TCAGCGCAGCCCTGGTTAAAAACAAGGTTTAATTTATAACCAAA TTTTCGTCCAATTTTTCTGTC CAATGCGGTACCCA3'
**SPR / GMSA / AFM**	
iterons	5'CCTGCGGTATTGACACTTGAGGGGCGCGACTACTGACAGATGAGGGGCG CGATCCTTGACACTTGAGGGGCAGAGTGATGACAGATGA GGGGCGCACCTATTGACATTTGAGGGGCTGTCCACAGGCAG3'
ssDNA DUE$_{oriV}$ top	5' CCACCGCTAACCTGTCTTTTAACCTGCTTTTAAACC AATATTTA TAAACCTTGTTTTTAACCAGGGCTGC3'
ssDNA DUE$_{oriV}$ bottom	5'GCAGCCCTG GTTAAAAACAAGGTTTATAAATATTGGTTTAAAA GCAGGTTAAAAGACAGGTTAG CGGTGG 3'
ssDNA DUE$_{oriV}$ bottom swap M1-R	5'CCACCGCATTG GACAGAAAAAACCTGCTTTTAAACCAATATTTA TAAACCTTGTTTTTAACCAGGGCTGC3'
ssDNA DUE$_{oriV}$ bottom swap M2-R	5'CCACCGCATTGGACAGAAAAATTGGACGAAAATAACCAATATTT ATAAACCTTGTTTTTAACCAGGGCTGC3'
ssDNA DUE$_{oriV}$ bottom swap R	5'CCACCGCATTGGACAGAAAATTGGACGAAAATTTGGTTATAAATTAAACC TTGTT TTTAACCAGGGCTGC3'
dsDNA DUE$_{oriV}$	5' CCACCGCTAACCTGTCTTTTAACCTGCTTTTAAACC AATATTTA TAAACCTTGTTTTTAACCAGGGCTGC3'
dsDNA pUC18 fragemnt	5'AGCTCACAATTCCACACAACATACGAGCCGGAAGCATAAAGTGTAAAG CCTGGGGTGCCTAATGAGTGAGCTAACTCACATTAATTGCGTTGCGCTC ACTGCCCGCTTTCCAGTCGGGAAACCTGTCGT 3'
ssDNA DUE$_{oriS}$ top	5'CCACGGGGATATTTTTATAATTATTTTTTTTATAGTTTTTAGATCTTCTTT TTTAGAGC3'
ssDNA DUE$_{oriS}$ bottom	5'GCTCTAAAAAAGAAGATCTAAAAACTATAAAAAAAATAATTATAAAAA TATCCCCGTG3'
oriV1	5'AAGCCGTGTGCGAGACACCGC3'
oriV2	5'AAAGACAGGTTAGCGGTGGCCG3'
oriS1	5'CTTCTGAGGGCAATTTGTCACAGG 3'
oriS2	5'CTTGGGGTTATCCACTTATCCA CG3'

Table S1. Oligonucleotides used in experiments.

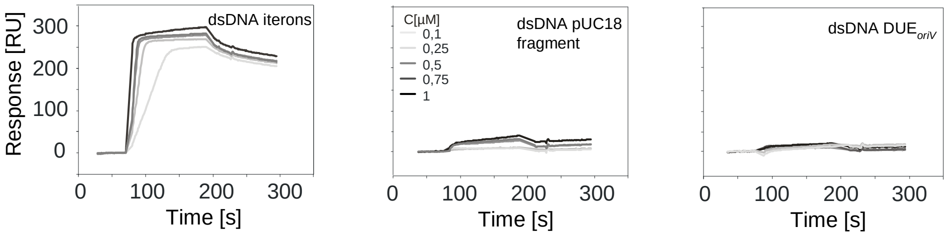

Fig. S1 Analysis of interaction TrfA protein with dsDNA. SPR analysis of TrfA protein interaction with dsDNA containing iterons, fragment of plasmid pUC18 or DUE region. Increasing amounts of protein (0.1, 0.25, 0.5, 0.75 and 1 µM) were run over the surface of a sensor chip with immobilized dsDNA.

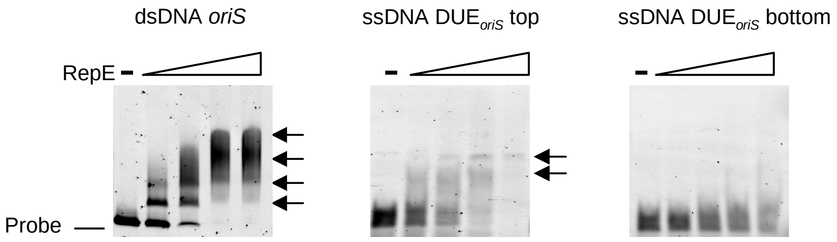

Fig. S2 RepE protein binds one specific strand of ssDNA in the DUE*oriS* of the origin. Electrophoretic mobility shift assay of RepE R118P was performed as described in Materials and Methods. Increasing amounts of RepE protein (5, 10, 15, 20 pmols) was incubated with 1 pmol fluorescently labeled dsDNA containing *oriS* origin region or ssDNA of DUE_{oriS}, top or ssDNA of DUE_{oriS} bottom strand, as noted. Black arrows indicate nucleoprotein complexes.

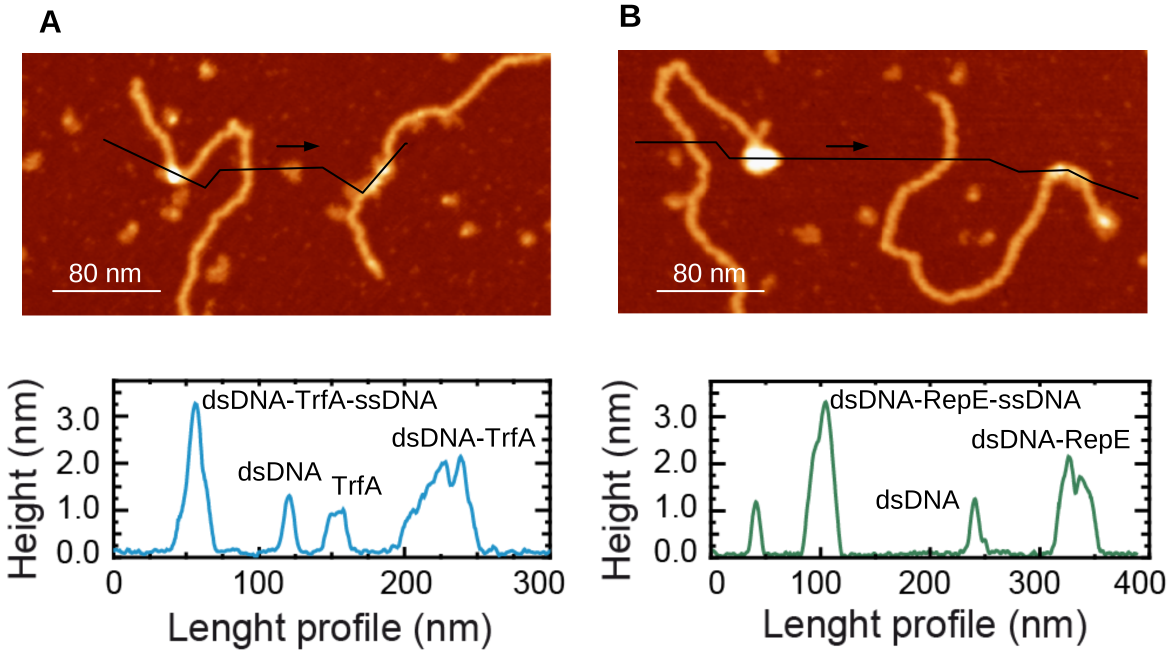

Fig. S3 AFM height characterisation of dipartite and tripartite Rep complexes. Rep protein, TrfA (A) or RepE (B), was incubated with dsDNA containing iterons and ssDNA of DUE sequence, and prepared for AFM imaging as described in *Methods*. Tripartite complexes (dsDNA-Rep-ssDNA) exhibited heights of around 3 nm and dipartite complexes (dsDNA-Rep) of around 2 nm. Two representative profiles for TrfA and RepE experiments are taken in the direction of the arrows and shown below AFM images. Colour scale in AFM images (from dark to white) is 0 - 2.5 nm.

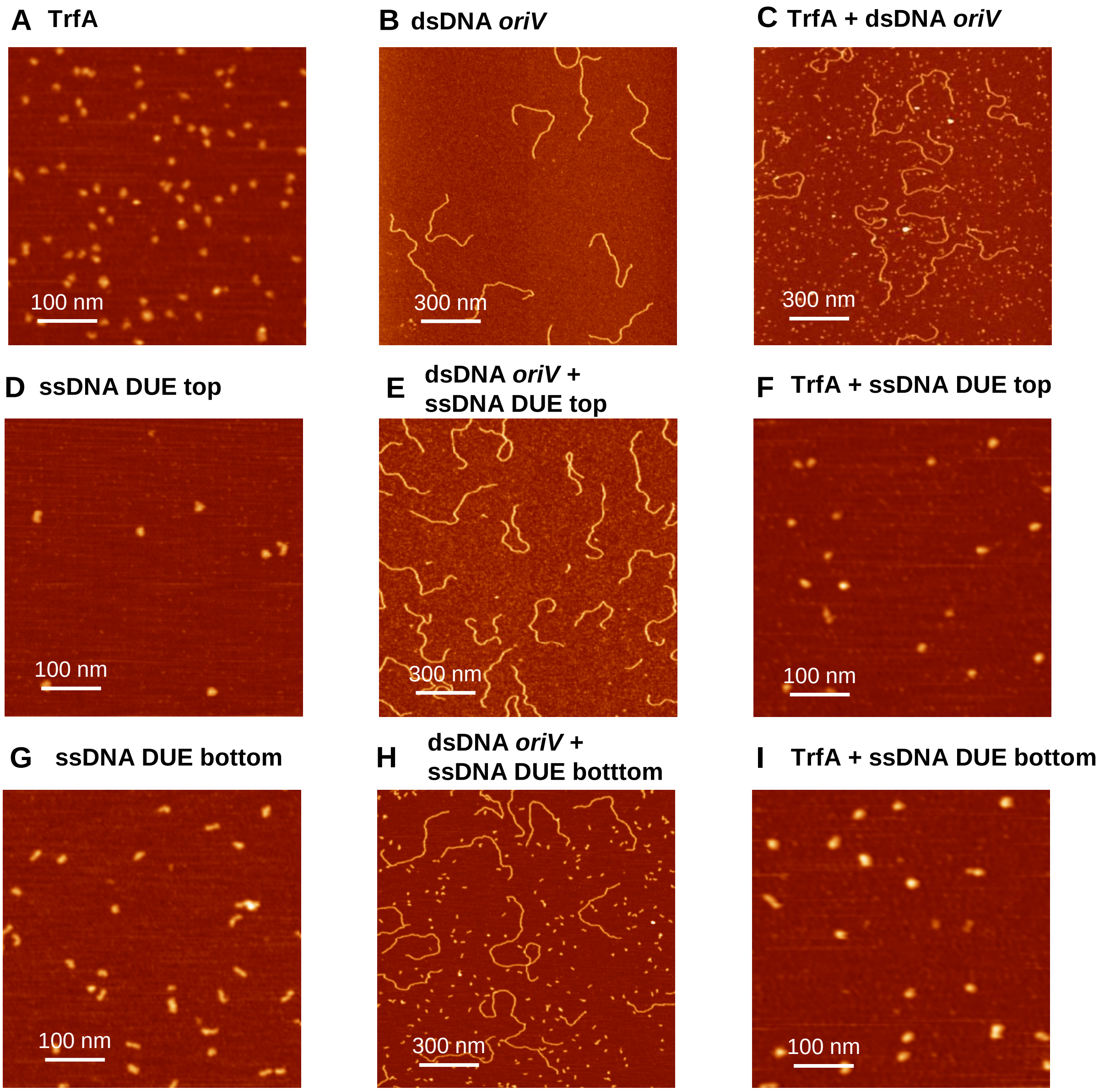

Fig. S4 Representative AFM images of TrfA protein and DNA. Protein TrfA, dsDNA or ssDNA fragments were incubated either alone (A, B, D, G) or with each other (C, E, F, H, I) and prepared for imaging as described in *Methods*.

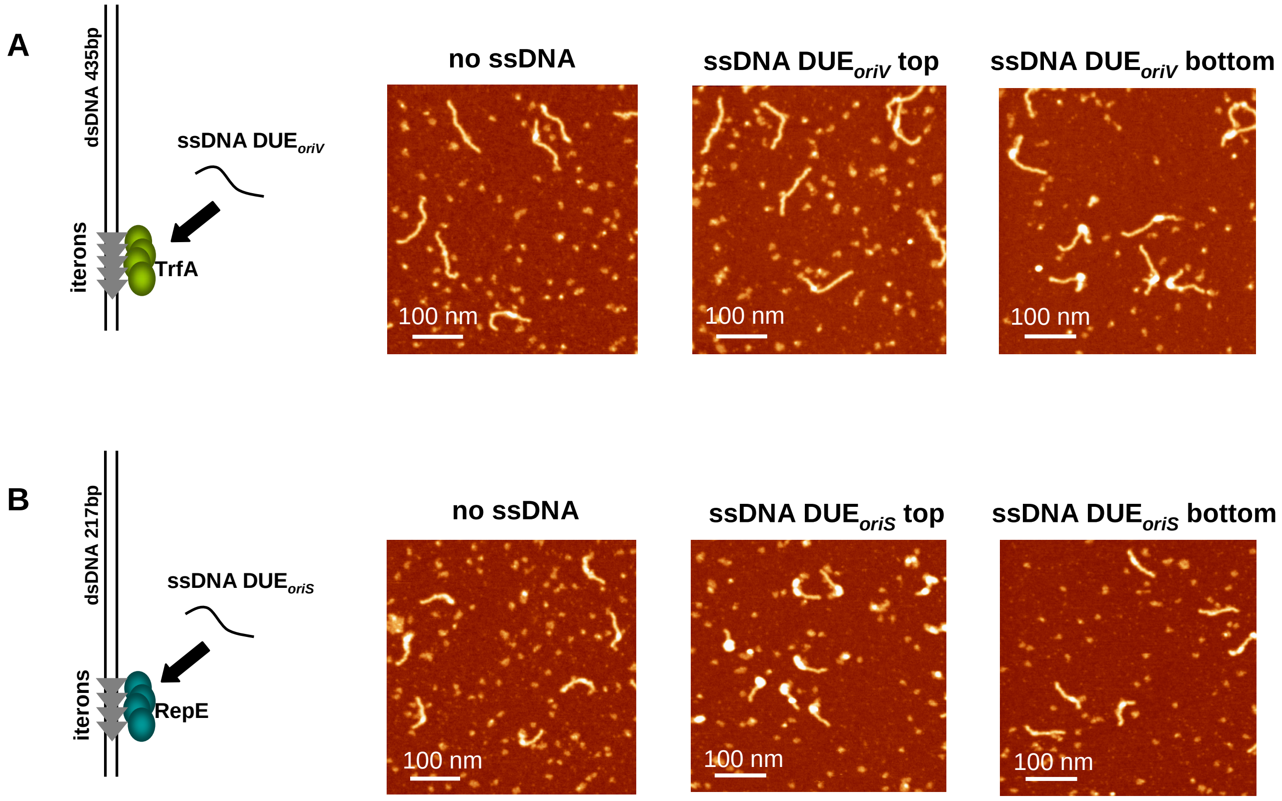

Fig. S5 Rep proteins form a complex consisting of linear dsDNA and ssDNA DUE strand. The formation of a tripartite nucleoprotein complex was imaged with AFM. The dsDNA fragments containing iterons sequence were incubated with Rep protein, TrfA (**A**) or RepE (**B**), and then ssDNA containing the DUE sequence of the top or bottom strand was added. After incubation the mixture was analyzed by AFM as described in Materials and Methods. The bright dot on dsDNA indicates the tripartite nucleoprotein complex.

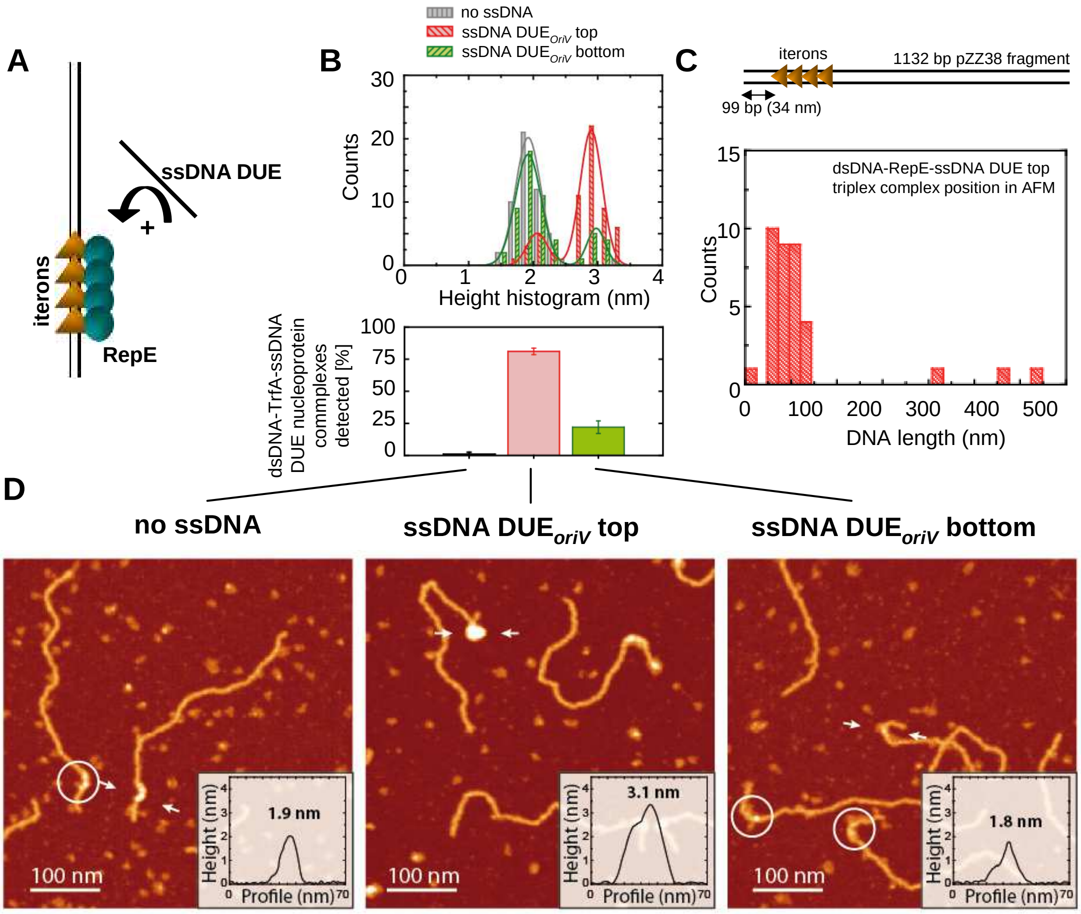

Fig. S6 AFM characterisation of RepE dipartite and tripartite complexes. (A) Scheme of the experiments: RepE protein was first incubated with linear dsDNA containing iterons and then added ssDNA with the sequence of the top or bottom strand of DUEoriS. (B) Top panel, height histogram of RepE dipartite and tripartite complexes: dsDNA-RepE (no ssDNA), dsDNA-RepE-ssDNA top (ssDNA DUE$_{OriS}$ top) and dsDNA-RepE-ssDNA bottom (ssDNA DUE$_{OriS}$ bottom); bottom panel, occurrence of tripartite complexes. (C) Top panel, cartoon showing the iterons position in the dsDNA substrate; bottom panel, histogram showing the binding position of tripartite complexes in the dsDNA substrate. (D) Representative AFM images of experiments. Characteristic profiles taken between white arrows are shown as insets. Other examples of complexes are encircled. Colour scale in AFM images (from dark to white) is 0 - 2.5 nm.

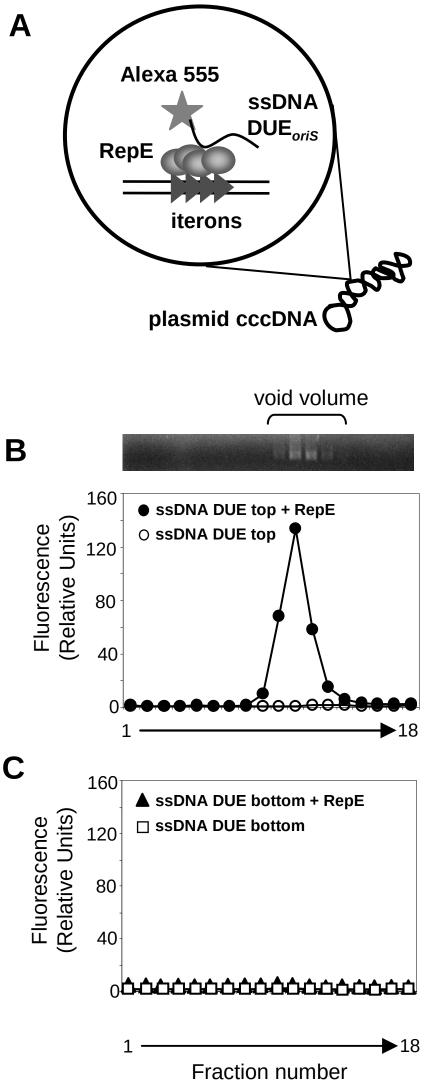

Fig. S7 RepE protein forms a complex consisting of plasmid cccDNA and ssDNA of the DUE$_{oriS}$ bottom strand. (**A**) The scheme of experiment. The supercoiled plasmid DNA containing iterons sequence was incubated with RepE protein and then fluorescently labeled ssDNA containing the DUE$_{oriS}$ sequence of the top (**B**) or bottom (**C**) strand was added. After incubation the mixture was separated using size exclusion chromatography. Fractions were analyzed for presence of plasmid cccDNA (top of the panel **B**) and fluorescently labeled ssDNA (graphs). Co-localization of the ssDNA and plasmid DNA in the void volume fractions indicate the tripartite complex formation.

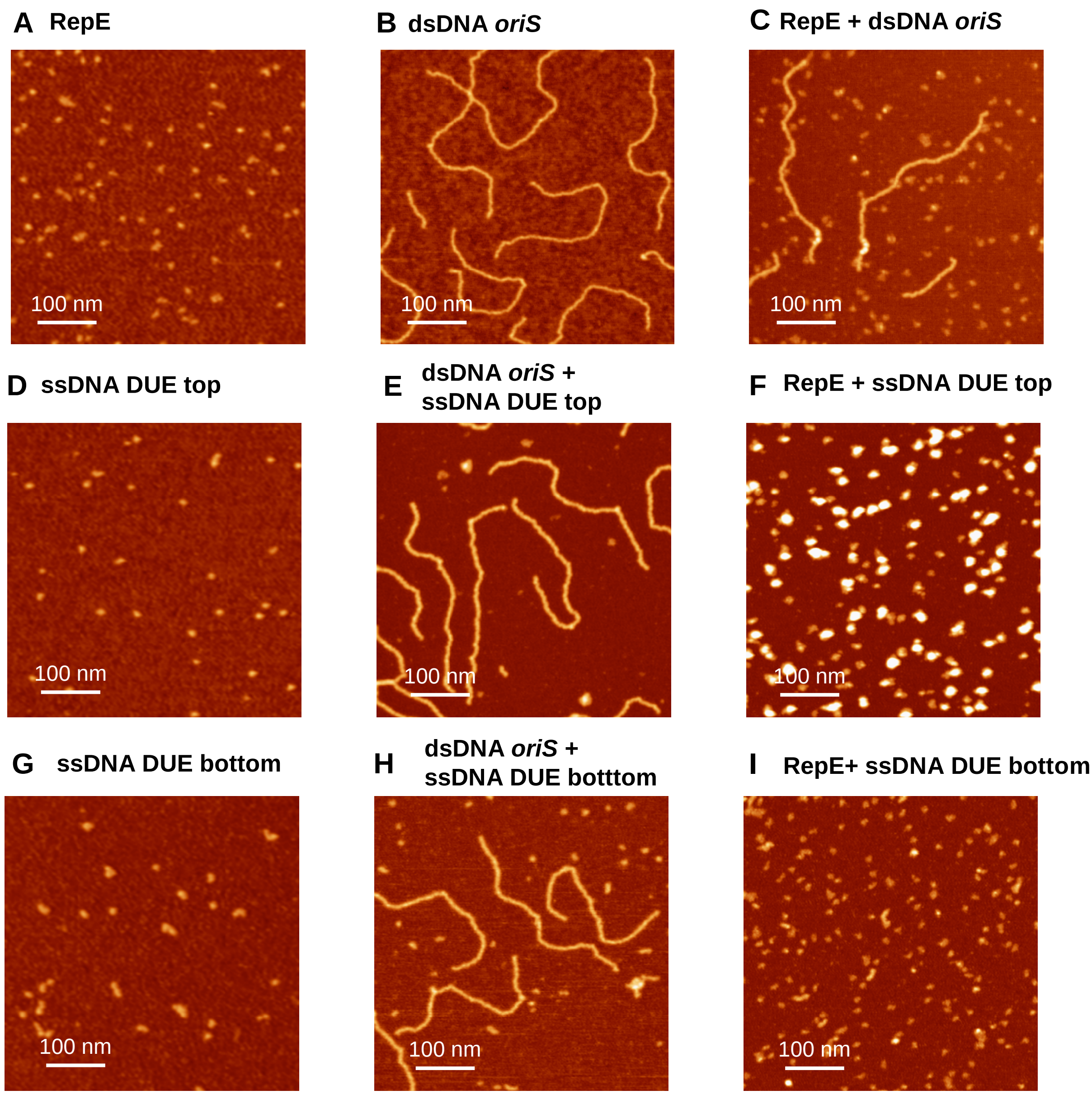

Fig. S8 Representative AFM images of RepE protein and DNA. Protein RepE, dsDNA or ssDNA fragments were incubated either alone (A, B, D, G) or with each other (C, E, F, H, I) and prepared for imaging as described in *Methods*.

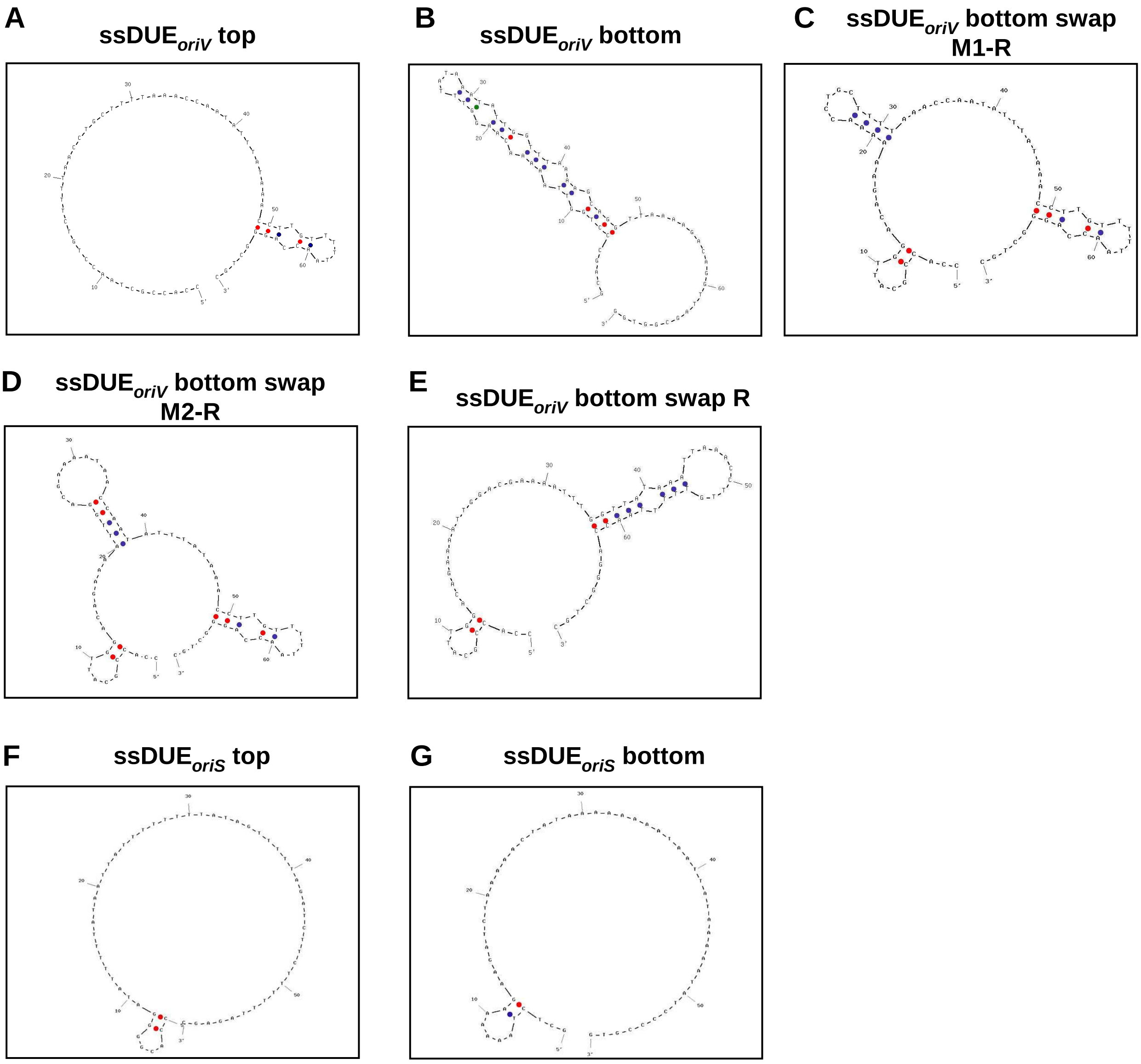

Fig. S9 Prediction of secondary structures formation within ssDNA DUE. The analysis of potential secondary structures formation (hairpin structures) was daone with OligoAnalyzer software (http://eu.idtdna.com/analyzer/Applications/OligoAnalyzer) based on Mfold algorithm (http://*mfold.burnet*.edu.au). Panels represent the most stable structures predicted for temperature 32°C and buffer contained 100mM NaCl. (A) ssDNA DUE$_{oriV}$ top, (B) ssDNA DUE$_{oriV}$ bottom, (C) ssDNA DUE$_{oriV}$ bottom swapM1-R, (D) ssDNA DUE$_{oriV}$ bottom swap M2-R (E) ssDNA DUE$_{oriV}$ bottom swap R, (F) ssDNA DUE$_{oriS}$ top, (G) ssDNA DUE$_{oriS}$ bottom
